# Auditory verbal hallucinations and continuum models of psychosis: A systematic review of the healthy voice-hearer literature

**DOI:** 10.1016/j.cpr.2016.10.010

**Published:** 2017-02

**Authors:** David Baumeister, Ottilie Sedgwick, Oliver Howes, Emmanuelle Peters

**Affiliations:** a Institute of Psychiatry, Psychology & Neuroscience, King's College London, Department of Psychology, London, UK; b NIHR Biomedical Research Centre for Mental Health, South London and Maudsley NHS Foundation Trust, London, UK; c Institute of Psychiatry, Psychology & Neuroscience, King's College London, Department of Psychosis Studies, London, UK

## Abstract

Recent decades have seen a surge of research interest in the phenomenon of healthy individuals who experience auditory verbal hallucinations, yet do not exhibit distress or need for care. The aims of the present systematic review are to provide a comprehensive overview of this research and examine how healthy voice-hearers may best be conceptualised in relation to the diagnostic versus ‘quasi-‘ and ‘fully-dimensional’ continuum models of psychosis. A systematic literature search was conducted, resulting in a total of 398 article titles and abstracts that were scrutinised for appropriateness to the present objective. Seventy articles were identified for full-text analysis, of which 36 met criteria for inclusion. Subjective perceptual experience of voices, such as loudness or location (i.e., inside/outside head), is similar in clinical and non-clinical groups, although clinical voice-hearers have more frequent voices, more negative voice content, and an older age of onset. Groups differ significantly in beliefs about voices, control over voices, voice-related distress, and affective difficulties. Cognitive biases, reduced global functioning, and psychiatric symptoms such as delusions, appear more prevalent in healthy voice-hearers than in healthy controls, yet less than in clinical samples. Transition to mental health difficulties is increased in HVHs, yet only occurs in a minority and is predicted by previous mood problems and voice distress. Whilst healthy voice-hearers show similar brain activity during hallucinatory experiences to clinical voice-hearers, other neuroimaging measures, such as mismatch negativity, have been inconclusive. Risk factors such as familial and childhood trauma appear similar between clinical and non-clinical voice-hearers. Overall the results of the present systematic review support a continuum view rather than a diagnostic model, but cannot distinguish between ‘quasi’ and ‘fully’ dimensional models. Healthy voice-hearers may be a key resource in informing transdiagnostic approaches to research of auditory hallucinations.

## Highlights

• Subjective perceptual experiences of voices appear similar in clinical and non-clinical groups.
• Healthy voice-hearers hear voices less frequently, with less negative content, more perceived control and from an earlier age.
• Healthy and clinical voice-hearers differ in beliefs about voices, voice-related distress, and affective difficulties.
• Healthy voice-hearers show more cognitive biases, psychiatric symptoms and functional impairments than healthy controls.
• Risk factors such as familial and childhood trauma appear similar between clinical and non-clinical voice-hearers.

## 1 Introduction

There is accumulating evidence that the experience of auditory verbal hallucinations (AVHs) is not uncommon in healthy individuals, and is not necessarily an indicator of psychopathology. A significant proportion of healthy individuals experience psychosis-like symptoms such as voice-hearing at some point in their lives; usually AVHs present as transient experiences, for example during childhood and adolescence, periods of bereavement or in the form of hypnagogic or hypnopompic false auditory perceptions (de Leede-Smith & Barkus, 2013). A recent meta-analysis estimated a median prevalence of 6% and median incidence of 1.2% of hallucinatory experience in the general population (Linscott & van Os, 2013). Notably, Linscott and van Os (2013) meta-analysis found that 20% of those who report psychotic experiences (including other phenomena such as delusional beliefs) go on to experience them persistently, 7.4% in the context of a psychotic disorder. These rates may be similar for AVHs specifically, as a recent cohort study of 1912 adolescents found that of the 5% who reported auditory hallucinations at baseline, they were still present in 27% two years later (De Loore et al., 2011). The term ‘healthy voice-hearers’ (HVHs) has been coined to describe individuals who experience persistent auditory verbal hallucinations, yet have no need for clinical care and do not suffer the significant distress this experience may cause in clinical populations (‘clinical voice-hearers’; CVHs). However, there remains uncertainty over how the two populations are related. The present systematic review aims to address such conceptual difficulties and provide a comprehensive overview of the currently available evidence.

The recent focus on AVHs in the healthy general population has arisen from a wider reconceptualization of psychosis and a shift from diagnostic to symptom-focused approaches. Classically, AVHs were defined as first-rank symptoms of schizophrenia (Schneider, 1959), as part of discrete, categorical models, i.e. those employed by diagnostic classification systems (Table 1; Model 1). However, these diagnostic models, although still employed in clinical practice, have been criticised for their lack of an empirical evidence-base (Bentall, 2003, Kaymaz and van Os, 2010, Linscott and van Os, 2010, Van Os, 2009). Transdiagnostic, symptom-focused approaches have been proposed both for psychosis (e.g., the transdiagnostic psychosis spectrum; van Os & Reininghaus, 2016) as well as wider mental health (e.g., the Research Domain Criteria project; Insel et al., 2010). AVHs are present in a range of mental health difficulties, including depression and anxiety, post-traumatic stress disorder, emotionally unstable personality disorder, and obsessive-compulsive disorder (Johns et al., 2014, Upthegrove et al., 2016, van Os and Reininghaus, 2016). Further, the impact and presentation of AVHs may differ within individuals in need for care, and there have been proposals to subtype AVHs in clinical research and practice (Smailes et al., 2015).

Conceptually, there has also been a marked shift from categorical models towards a continuum view of psychotic symptoms and anomalous experiences that extends not just across diagnostic categories but also into the (healthy) general population. This has long been proposed by researchers such as Claridge (1994) and Bentall (2003), and has gained considerable epidemiological support (Linscott and van Os, 2013, Linscott and van Os, 2010, van Os et al., 2009). According to the continuum model, HVHs are situated on a continuous dimension between CVHs and non-voice-hearing healthy individuals (healthy controls; HCs) in terms of their anomalous experiences, but without crossing the threshold for need for care. However, different conceptualisations of the continuum model exist in the literature (see Table 1; Models 2 & 3). Claridge (1994), Claridge & Beech (1995) has differentiated between ‘quasi-dimensional’ (Table 1; Model 2) and ‘fully dimensional’ (Table 1; Model 3) models. In the former, the continuum describes disease severity; it is assumed that psychotic experiences and distress are part of the same dimensions and that psychotic experiences are ultimately indicative of a psychobiological abnormality but simply in attenuated form. It is further assumed that only a small proportion of the general population has a predisposition for such experiences. In a fully dimensional model, however, the continuum of anomalous experiences may be largely independent from the continuum of clinical distress or need for care, and makes no prediction regarding the outcome of psychotic experiences. The propensity for such experiences is distributed in the general population as part of normal individual differences and only in extreme forms necessitates care. Such a conceptualisation is more in line with viewing voice-hearers without need for care as being truly “healthy”, rather than merely “subclinical”.

However, these conceptualisations may still be over-simplistic (Kaymaz and van Os, 2010, Linscott and van Os, 2010). Linscott and van Os (2010) carried out a systematic review and meta-analysis of primarily epidemiological data on what they refer to as the ‘extended phenotype model’. Their results suggest that there is evidence for continuity of symptoms, based on the high incidence and prevalence rates of psychotic experiences in the general population compared to the actual rate of clinical psychotic disorders. However, they also found evidence for a dichotomous distribution of individuals who have a liability to schizotypal traits from individuals who do not. These mixed findings suggest the possibility that the psychosis continuum may encompass two latent, discontinuous subgroups, leading to a hybrid conceptualisation of quasi- and fully-dimensional models. Current evidence further suggests that psychosis is a complex multifactorial construct, with individual symptoms or characteristics: a) lying on individual continua (Russo et al., 2014, Van Os, 2009); b) showing differing prevalence rates and causal factors (Wigman et al., 2011b, McGrath et al., 2015); c) having differing implications for a need for care or clinical risk (Wigman et al., 2011b, Kaymaz et al., 2012); and d) demonstrating varying correlational or predictive relationships with other symptoms (Wigman et al., 2011a, Bell et al., 2008). Most recently, van Os & Reininghaus (2016) have proposed a transdiagnostic psychosis spectrum in which psychotic symptoms in the general population are continuous with clinical psychotic disorders, but can nonetheless present independently. This conceptualisation encompasses both specific psychosis factors (e.g., positive symptoms) as well as nonspecific associations with psychopathology (e.g., affective dysregulation), and the combination of these two underlying constructs then becomes critical in leading to a need for care.

In an editorial aiming to stimulate the continuum debate, David (2010) suggests that the continuum hypothesis should be taken as the null hypothesis, and the present review examines whether there is evidence to refute it in relation to AVHs specifically. The focus on AVHs allows investigation of the psychosis continuum in the context of a specific phenomenon of the psychosis dimension that presents both across health-pathology and across different types of pathology. Assessing whether the available research on HVHs has produced results congruent with the current evidence on the psychosis continuum can attest to its relevance and add to its validity. Indeed, Johns et al. (2014) call on research to investigate the role of the quasi- and fully-dimensional continua in AVHs in healthy individuals. In turn, the psychosis continuum models provide an important context to determine to what extent HVHs are “healthy” and are likely to remain so. For instance, whilst HVHs may present as currently healthy, the transdiagnostic extended phenotype model presented by van Os & Reininghaus (2016) notes the temporal continuity of psychotic experiences with clinical disorders, i.e., HVHs may be at greater risk of psychotic disorders long-term. Furthermore, examination of the relationships between AVHs in healthy populations and other symptom dimensions and characteristics relevant psychosis, such as affective difficulties, risk factors, or neurobiological substrates, may be valuable for the understanding of AVHs and need for care in clinical populations.

Whilst the reviewed continuum conceptualisations relate to psychosis or schizotypal personality traits across the wider population, rather than the specific phenomenon of auditory hallucinations, their relevance to AVHs in healthy individuals is inferred here. Similarly, whilst still relevant, many studies in the HVH literature were not carried out with the continuum hypothesis in mind and are thus integrated into an overarching framework to consider this literature. According to the diagnostic model, benign AVHs should be highly dissimilar as an experience to those found in CVHs, and HVHs and HCs should be indistinguishable on almost all parameters (e.g., risk factor exposure). According to the quasi-dimensional model, HVHs will be on a middle-point between CVHs and HCs on almost all parameters, including need for care and voice-distress. In such a model, increases in the occurrence of psychotic experiences would be associated with increased need for care. Lastly, a fully-dimensional model would predict that the occurrence of AVHs is largely unrelated to need for care, and HVHs should not be at greater risk of distress than HCs. Other parameters should vary at random. However, according to the more recent epidemiological conceptualisation of extended, transdiagnostic phenotypes with latent subgroups, the available evidence would be expected to support both quasi- and fully-dimensional models to a similar degree. Thus, the present review has two main hypotheses: firstly, the evidence will be incompatible with the diagnostic model; secondly, the evidence will provide support for both quasi- and fully-dimensional models, depending on methodology used and sample characteristics of the study.

Several narrative reviews have been published on AVHs in healthy populations (Badcock and Chhabra, 2013, Badcock and Hugdahl, 2012, de Leede-Smith and Barkus, 2013, Johns et al., 2014, Larøi, 2012). However, these tend to be broader (e.g. inclusive of prodromal populations), or more theoretical or narrow in their discussion (e.g. of neurocognitive mechanisms) than the focus of the present review. Moreover, by their narrative nature, they are more vulnerable to bias than the systematic approach undertaken here. The present systematic review aims to: give a comprehensive overview of the phenomenon of persistent AVHs in healthy adult populations; consider the evidence for models of the psychosis continuum in the context of AVHs; and identify areas where future research is needed.

## 2 Methods

### 2.1 Search strategy

**Fig. 1:**
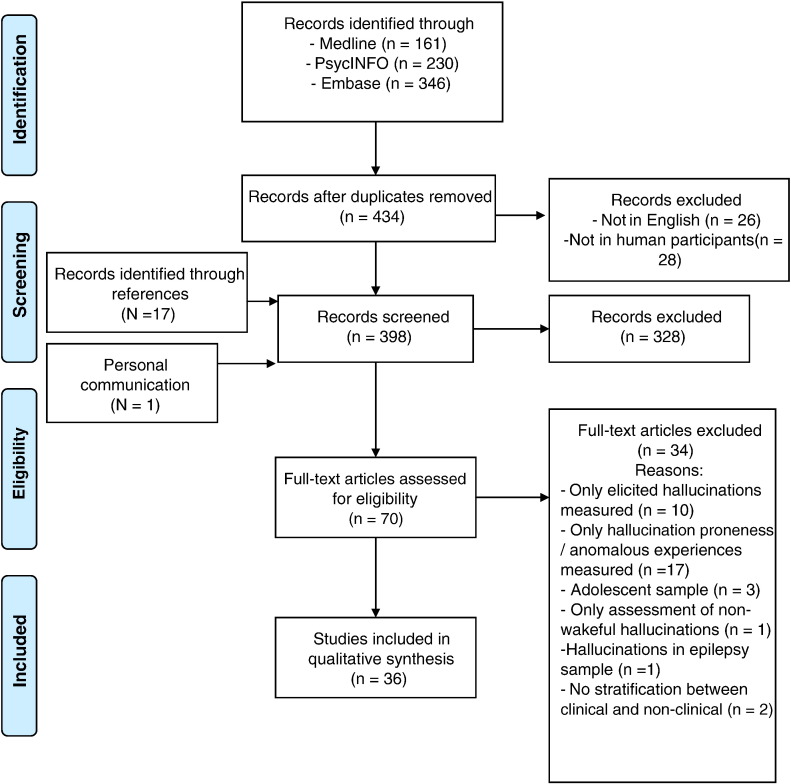
PRISMA diagram of the study selection.

A systematic review of the literature was performed using PsycINFO, EMBASE, and Medline for the subject headings “auditory hallucination*” and “voice hear*” cross-referenced separately with the terms “healthy”, “no need for care” and “non-clinical”. The literature review was performed in February 2016. Articles were limited to research in human participants, and published in English language. The initial search produced 230 on PsycInfo, 346 on Embase and 161 on Medline (see Fig. 1). Additionally, 17 papers were identified through search of references in identified papers. One additional paper was identified through personal communication with the authors (Jacobsen et al., Under Review). The following criteria were used for exclusion and inclusion into the review:

Exclusion criteria:

• Only voice-hearers with a clinical diagnosis of a psychotic disorder or other conditions associated with AVHs (e.g. PTSD, epilepsy)
• Only hallucination-proneness assessed (e.g. Launay-Slade Hallucination Scale (LSHS; Launay & Slade, 1981) scores) and no reporting of current AVHs
• Childhood and adolescent samples
• General assessment of anomalous experiences only
• Elicited hallucinatory experiences (e.g. signal detection tasks or through hypnosis)
• Drug-induced hallucinations
• Non-verbal hallucinations

Inclusion criteria:

• Studies with a sample of individuals without clinical diagnoses who report hearing voices but no related distress
• Articles published in English language

### 2.2 Selection

After exclusion of duplicates, articles not published in English language, and studies not including human participants, 398 article titles and abstracts were scrutinised for inclusion into the review. Seventy appropriate articles were identified for full-text analysis, of which 36 met criteria for inclusion. Full-text analysis and data extraction were carried out independently by two authors (DB & OS), and any inconsistencies were discussed until consensus was reached. Notably, several of the identified studies (from the Dutch (Utrecht) group, marked in Table 2) included the same or overlapping samples, however often with slightly different numbers of participants and different main outcome measures. Ineligible articles (n = 34) were excluded for the following reasons: only hallucination proneness/anomalous experiences measured (n = 17); only elicited hallucinations measured (n = 10); adolescent sample (n = 3); only assessment of non-wakeful hallucinations (n = 1); hallucinations in epilepsy sample (n = 1); no stratification for need for care (n = 2) (see Fig. 1). Studies where samples were selected purely on the basis of proneness to hallucinations (e.g., using a total score on the LSHS) were excluded as such measures may include a) non-AVH hallucinations and b) transient experiences. However, studies that used individual AVH-specific LSHS items (e.g., “In the past I have had the experience of hearing a voice and then found no one was there”) as part of their inclusion criteria were included (see Table 2), if they satisfied the criterion of ‘reporting of current AVHs’.

Study characteristics are presented in Table 2. The results presented below are organized with a focus on specific characteristics that have emerged from the literature, rather than by their congruence with the explanatory models evaluated here, which is returned to in the discussion. The structure of the results is aimed at aiding the reader interested in discrete aspects of HVH research, and improving reading experience and accessibility. Results are presented by the following characteristics: voice phenomenology, their impact and appraisal, mood disturbances, impairment and functioning, related psychotic phenomena, cognitive functioning, neuroimaging, trauma exposure and familial risk.

## 3 Results

### 3.1 Methodology

Out of the 36 studies reviewed, 17 were drawn from the Dutch (Utrecht) sample of HVHs, comparing them to HCs and/or CVHs (studies from this cohort are marked with an asterisk). These studies employed the same selection and screening criteria, which were amongst the most stringent (see Table 2). Although these studies generally had different main outcomes, some of the basic data such as voice phenomenology were assessed in samples recruited from the same cohort, albeit with slightly different participant numbers in each. Therefore separate publications may report the same finding, confounding any cumulative strength of evidence by the shared participants across studies. Nevertheless, these studies had different clinical and/or healthy control samples, and did not always report the same results on the same measure. Therefore they are still reported as individual findings, but with an indication (*) that they belong to one cohort (see Table 2, Table 3).

Sample sizes differed considerably depending on methodology employed across all 36 studies. As would be expected, studies relying largely on questionnaire-based data had larger sample sizes than studies using neuroimaging or qualitative assessments. Although a priori matching across samples for at least one variable occurred in a sizeable minority of studies, primarily handedness, gender and/or age, several studies reported that samples did not match on education. Moreover, it should be noted that both CVH and HVH within and between individual studies are likely to show considerable degrees of heterogeneity, both due to differences in recruitment strategies and sources, as well as differing diagnoses in CVHs.

### 3.2 Phenomenology

Twenty-seven of the reviewed studies reported on the phenomenology of voices in some capacity, 14 of which were from the same cohort. Phenomenological similarities and differences in AVH are presented in Table 3, and summarised below, in a subset of 17 studies that compared the major phenomenological characteristics of AVHs in HVHs and CVHs. Finally, Daalman et al. (2016*) report that AVHs in non-clinical samples show a high level of persistence, with continued experience of AVHs in 86.4% of their sample at 5-year follow-up.

#### 3.2.1 Age of onset

Five out of six publications comparing age of AVH onset reported an earlier age in HVHs than CVHs (Daalman et al., 2011, De Weijer et al., 2013, Honig et al., 1998, Sorrell et al., 2010), with age of onset in HVHs typically occurring between late childhood and early adolescence (Daalman et al., 2011, Linden et al., 2011, Sommer et al., 2010a, van Lutterveld et al., 2010). However, Kråkvik et al. (2015) did not find a significant difference in age of onset between CVHs and HVHs.

#### 3.2.2 Frequency and duration of voices

Fourteen out of 15 studies reported a lesser frequency of voice-hearing in HVHs, with only one study failing to find a significant difference. Similarly, eight out of ten studies reported a lesser duration of hallucinatory episodes in HVHs, although two found no difference between HVHs and CVHs.

#### 3.2.3 Perceptual qualities

Eleven studies compared the loudness of voices between HVHs and CVHs, with 8 finding no significant difference, two reporting quieter voices and one reporting louder voices in HVHs. Similarly, in 10 studies all but one reported that the perceived location of voices did not differ between HVHs and CVHs, with only one reporting that HVHs were more likely to perceive them as located inside the head (Leudar, Thomas, McNally, & Glinski, 1997). There is some evidence that HVHs perceive their voices with less clarity than CVHs (Cottam et al., 2011, Lawrence et al., 2010), but similar rates report their voices as indistinguishable from real voices (Moritz & Larøi, 2008).

#### 3.2.4 Voice identities

Three out of four studies reported that HVH heard fewer different voices, particularly those commenting in the 3rd person. The majority of HVHs appear to hear one voice, although a sizeable minority hear multiple voices, with more than 10 in 5.4% of HVHs (Lawrence et al., 2010). According to Sommer, Daalman et al. (2010*) 18% of HVHs reported commenting voices, and 11% heard voices speaking with each other; similarly, Peters et al. (2016) reported fewer commenting or conversing voices in HVHs compared with CVHs. Leudar et al. (1997) reported that both CVHs and HVHs are addressed by voices directly, and voices commonly sound like individuals known to the voice-hearers; whilst voices in the clinical group are more frequently those of public figures or supernatural characters, HVHs are more likely to identify voices as similar to themselves or family members (Leudar et al., 1997). However, Kråkvik et al. (2015) found no differences in the voice identities reported by CVHs and HVHs. Further, Sorrell et al. (2010) reported that gender and identity of AVHs does not appear to differ between groups. Religious groups more frequently identified their voices to be religious entities, however HVHs more often heard “God” and rarely “the Devil”, whilst CVHs more often heard “the Devil” but rarely “God” (Cottam et al., 2011).

#### 3.2.5 Content

Of the 14 studies comparing HVHs and CVHs, all reported lower levels of negative voice content and emotional valence in HVHs. Indeed, in one sample 71% of HVHs had never experienced negative voice content (Sommer, Daalman et al., 2010*). Similarly, voices in religious HVHs mostly have mixed or neutral content, whereas religious CVHs mostly hear mixed and negative content (Cottam et al., 2011). However, Beavan and Read (2010) found that, in a sample of CVHs and HVHs that were not formally stratified by clinical status, no participants had experienced positive voice content only. In a small qualitative study, Leudar et al. (1997) found that directive voices in CVHs frequently issued commands to carry out specific actions or violent acts, but in HVHs they more commonly “gave advice” on a particular course of action or mundane activities. HVHs heard significantly fewer negative evaluative comments about themselves, including their own thoughts (Honig et al., 1998), but heard significantly more comments evaluating others. This was also reported in the larger sample of Kråkvik et al. (2015), where HVHs were less likely to hear voices commenting on them. Whilst there was no difference in commanding voices, CVHs were more compliant with and swayed by commands. Interestingly, Varese, Tai, Pearson, and Mansell (2016) identified personal goals (e.g., being a confident person) as a substrate of voice content: in the majority of both CVHs and HVHs, personal goals of participants matched the content of the voices they experienced.

### 3.3 Voice impact and appraisal

#### 3.3.1 Distress and control

As would be expected, out of the 23 studies investigating distress all reported that voice-hearing in HVHs was associated with little to no voice-related distress, and/or that voice distress was significantly higher in CVHs. Comparing HVHs and CVHs, 10 studies found that HVHs reported greater control over voices, with only two studies finding the same level of control in HVHs and CVHs. Indeed, one study reported that healthy status was significantly predicted by high control over voices, low frequency of voices, age of onset before age 16, and predominantly positive voice content (Daalman et al., 2011, Daalman et al., 2011). Need for control and low perceived control were also found to predict voice-distress by Hill, Varese, Jackson, and Linden (2012), whilst Beavan and Read (2010) reported that negative emotional responses were predicted by negative voice content, more voices talking or arguing with each other, commenting on the individual, talking for longer periods, and taking over thoughts of the individual (Beavan & Read, 2010), as well as disturbing contact with others (Kråkvik et al., 2015). CVHs are significantly more afraid of voices than HVHs, and see voices as troublesome and disturbing daily life (Honig et al., 1998). Interestingly however, one study indicated that despite negative elicited emotions being more likely to be reported by the CVH group, there was no significant difference in positive emotions elicited by AVHs in CVHs and HVHs (Kråkvik et al., 2015). Nonetheless, more than 90% of HVHs report no disturbance to their life by AVHs (Sommer, Daalman et al., 2010*), and all six studies comparing the disruptive impact of voices between HVHs and CVHs reported less disruption in HVHs.

#### 3.3.2 Beliefs about voices

Out of the eight studies comparing beliefs of origin between HVHs and CVHs, six found that HVHs were more likely to attribute the voices to external origins, whereas two found no significant difference between the groups. All of the six studies assessing beliefs about voices indicate that HVHs have significantly less negative beliefs about voices, which is associated with more positive voice impact. Hill et al. (2012) reported that CVHs scored higher than HVHs on negative beliefs about worry and need for control of thoughts. Voice-related distress was significantly associated with negative beliefs about uncontrollability and danger of voices. Lawrence et al. (2010) found that, compared to scores from a previously published sample of CVHs, HVHs had significantly lower beliefs of malevolence, omnipotence and resistance towards voices, but higher scores of benevolence and engagement with voices. Levels of distress correlated with malevolence, omnipotence and resistance. Higher frequency was associated with higher levels of depression, anxiety, malevolence, omnipotence and resistance. Andrew, Gray, and Snowden (2008) found that CVHs were more likely to appraise their voices as malevolent, which was predictive of depressive symptoms, and were more likely to use resistant coping strategies. Similarly, Kråkvik et al. (2015) found that CHVs were more likely to try to actively ignore voices, including command hallucinations (Leudar et al., 1997), and to try to understand them or argue with them, whilst a greater proportion of HVHs than CVHs were likely to do nothing in response to AVHs. Further, CVHs who begged voices to keep silent reported increased AVH intensity (Kråkvik et al., 2015). In turn, Peters et al. (2016) reported that HVHs were more likely to be accepting of their voices, and adopt a mindful response style compared with CVHs. Qualitative data suggest that in HVHs, the initial reaction is marked by resistance, which is associated with increased intrusiveness, but eventually engagement (i.e. understanding and acceptance of experience) mitigates distress (Taylor & Murray, 2012). Sorrell et al. (2010) reported that HVHs related to their voices with less distance. Voice dominance, intrusiveness and hearer distance were significantly correlated with distress. However when controlling for beliefs of malevolence and omnipotence, the association of distress and relating variables lost significance. Recently, Daalman et al. (2016*) provided evidence that attitudes towards AVHs can be susceptible to fluctuations, with beliefs about voices changing in 15.7% of HVHs at a 5-year follow-up.

#### 3.3.3 Spiritual frameworks

All four studies reporting on spiritual or religious frameworks showed that these are more frequently employed by HVHs, with generally positive perceived impact. Daalman, Boks et al. (2011*) reported that HVHs more frequently endorsed unspecific external or spiritual explanations, whereas CVHs more frequently explained voices to be other (living) people, god, demons/devil or implanted devices. In their comparison of religious HVHs to religious and non-religious CVHs, Cottam et al. (2011) found that religious HVHs more often experienced AVHs as a positive but never a negative power, whereas most clinical participants (both religious and non-religious) appraised them as a negative power. Similar findings were reported by Davies, Griffin, and Vice (2001), with religious HVHs having significantly more positive perceptions of voices than non-religious HVHs and CVHs, respectively. In a qualitative study of HVHs recruited as psychic mediums, initial voice distress was mitigated by engagement with voices and integration into a spiritual framework (Taylor & Murray, 2012).

### 3.4 Mood disturbances

Three studies formally assessed mood disturbances in HVHs, comparing them to CVHs but not to HCs, with all three finding higher rates of emotional difficulties in the CVHs. Andrew et al. (2008) reported greater rates of depression and anxiety in CVHs compared with HVHs. Similarly, Sorrell et al. (2010) reported significantly greater depression scores in CVHs than HVHs. Lawrence et al. (2010) found that scores for anxiety and depression were significantly lower in HVHs than for 71 CVHs in an external study sample. However, a number of studies (see Table 2) stipulated an absence of diagnosable affective disturbances as part of their inclusion criteria for HVHs. Nevertheless, Sommer, Daalman et al. (2010*) additionally reported on previous single or recurrent depressive episodes in full remission, and found that HVHs and HCs did not differ in their prevalence. The only study that compared depressive and anxiety symptoms in CVHs, HVHs and HCs reported significant group differences between all groups (Kråkvik et al., 2015) with CVHs having the highest scores and HCs having the lowest scores. Indeed, the HVHs in this sample were also significantly more likely than HCs (but less likely than CVHs) to have consulted a professional or received treatment for mental health problems unrelated to voice-hearing, and there is evidence that AVHs are associated with anxiety in the general population (Fleming & Martin, 2009). Woods, Jones, Alderson-Day, Callard, and Fernyhough (2015)’s survey data showed that voice-hearers who had not previously received a psychiatric diagnosis were less likely to associate their voices with fear or depression. Most recently, Daalman et al. (2016*) provided 5-year follow-up data on the mental health of their sample of HVHs as well as HCs. Eighty-one individuals with AVHs and 49 HCs were included, representing 78.6% and 81.7%, respectively, of the original participants. Five individuals with AVHs had transitioned to psychosis yet none of the HCs had developed psychosis. This difference was only at trend-level, and disappeared when individuals with previous depressive episodes who were in remission at baseline were excluded. However, they also found that 39.5% of their previously healthy voice-hearers had developed the need for mental healthcare, significantly more than the 12.2% of the healthy control group, even after exclusion of individuals with depression in remission at baseline. Regression analyses revealed that this need for mental healthcare was predicted by total distress of AVHs and depression in remission, but not global functioning, schizotypy, familial psychosis, childhood trauma, or AVH frequency, control, emotional valence or age of onset.

### 3.5 Impairment and functioning

Seven of the identified studies, all of which stem from the same Dutch cohort, reported on the potential impairment of HVHs, suggesting some impairments in global functioning that may be lesser than those of CVHs, yet greater than in HCs. Sommer, Daalman et al. (2010*) found that global functioning was significantly lower in HVHs than HCs, and was predicted by genetic loading (i.e. prevalence of familial psychiatric disorder). This was corroborated by Diederen et al. (2010*) and van Lutterveld et al. (2014*), who found that CVHs, HVHs and HCs all differed significantly from each other in their global functioning, with CVHs scoring the worst, and HVHs scoring better than CVHs yet worse than HCs. Additionally, Diederen et al. (2010*) reported that CVHs showed reduced global functioning compared to HVHs. Howes, Shotbolt, et al. (2013*) and Diederen et al. (2013*) reported that HVHs showed no impairment in global functioning, but did not compare the results of HVHs to HCs. De Weijer et al. (2013*) reported global functioning scores as part of their demographic variables, showing lower scores in functioning of HVH compared to HCs, but did not report on the statistical significance of this difference. Based on the reported data, we conducted a two-tailed *t*-test assuming unequal variances for a more conservative estimate, showing that this difference was significant (*p* = 0.005, *t* = 2.95).

### 3.6 Related psychotic phenomena

Six studies investigated other psychotic experiences in HVHs. Sommer, Derwort et al. (2010*) investigated thought disorder in CVHs, HVHs and HCs using a thought and language index and a thematic apperception test. Impoverishment of language was almost exclusively present in CVHs. Disorganization scores were significantly lower in HCs than HVHs and CVHs, but HVHs were significantly less disorganised than CVHs. Additionally, Sommer, Daalman et al. (2010*) reported that there was greater preoccupation with, and conviction of, delusional ideation in HVHs than HCs. Hill et al. (2012) found that CVHs scored higher than HVHs on positive symptoms, negative symptoms and symptoms of general psychopathology. HVHs did not differ significantly from HCs on negative symptoms and general psychopathology, but scored higher on positive symptoms, which lost significance when the hallucination item was excluded. Sommer, Daalman et al. (2010*) reported greater schizotypy scores in HVHs compared to HCs, with significant elevations on all subscales including non-positive dimensions. Interestingly, schizotypy scores, alongside genetic family loading and number of years of education, predicted global functioning. Higher schizotypy was also observed amongst HVHs compared to HCs in another study (van Lutterveld et al., 2014*), however schizotypy scores in one HVH group were similar to published general population estimates (Howes et al., 2013, Howes et al., 2013). Further, the majority of HVHs experience other hallucinatory experiences, most commonly in visual, olfactory and tactile sensory domains (Sommer et al., 2010a, Peters et al., 2016).

### 3.7 Cognitive biases

Two studies investigated the presence of cognitive biases in HVHs. Daalman et al. (2013*) compared CVHs, HVHs and HCs on cognitive biases for psychosis, including jumping to conclusions (reaching conclusions with limited information), intentionalising (suspecting ill intent in the actions of others), catastrophizing (endorsing the worst possible outcome of a situation), dichotomous thinking (appraising situations in extremes rather than gradients of good and bad) and emotional reasoning (emotion-driven reasoning, such that appraisals are based on internal emotional states). HCs had significantly lower cognitive biases scores than both HVHs and CVHs, and HVHs had lower scores than CVHs. However, there were different patterns depending on which type of bias/vignette content was examined: HCs and HVHs scored significantly lower than CVHs on intentionalising, catastrophising, dichotomous thinking and jumping to conclusions subscores, and did not differ from each other; whilst both AVH groups scored significantly higher on the emotional reasoning subscale compared to HCs and did not differ from each other. CVHs scored significantly higher on vignettes with threatening themes than both HCs and HVHs, who did not differ from each other. In contrast, both CVHs and HVHs scored significantly higher on vignettes with themes relating to anomalous perceptions compared with HCs, and did not differ from each other. Emotional (voice-distress and emotional valence) as well as cognitive (beliefs about origin, control and disruption) interpretations of AVHs were significant predictors of cognitive bias scores. Similarly, Jacobsen et al. (Under Review) found evidence for a more overgeneral autobiographical memory bias in CVHs compared to HVHs and HCs. Moreover, voice-specific autobiographical memory was more overgeneral in CVHs than in HVHs.

### 3.8 Cognitive functioning

A total of five studies, all but one stemming from the Dutch cohort, investigated cognitive functioning in HVHs, suggesting few significant differences compared to HCs. These include more errors in top down semantic expectation when compared to HCs (Daalman, Verkooijen, Derks, Aleman, & Sommer, 2012*). Moreover, auditory acuity appears somewhat lower in HVHs than HCs (Kompus et al., 2013). Similarly, some cognitive functions, mainly in the verbal domain, have been demonstrated to be significantly lower in HVHs compared to HCs (Daalman, van Zandvoort, et al., 2011*). Notably however, cognitive functions of HVHs were still within normal ranges. Interestingly, follow-up analysis by Begemann et al. (2016*) suggested that differential verbal inhibition, as measured by the Stroop paradigm (but no other cognitive measures), in HVHs vs HCs is fully explained by childhood trauma. A paced verbal fluency task has shown to be equivalent between HVHs, CVHs and HCs (Diederen et al., 2010*). Further, there were no differences observed at the behavioural level between HVHs and HCs on a test of effortful attention, as assessed via the oddball paradigm (van Lutterveld et al., 2010*).

### 3.9 Neuroimaging

A total of 9 studies used neuroimaging to investigate HVHs, with methodologies ranging from electroencephalography (EEG), functional magnetic resonance imaging (fMRI), structural MRI, diffusion tensor imaging (DTI) as well as positron emission tomography (PET) (Table 4; De Weijer et al., 2013, Diederen et al., 2013, Diederen et al., 2010, Diederen et al., 2012, Howes et al., 2013, Howes et al., 2013, Kompus et al., 2013, Linden et al., 2011, van Lutterveld et al., 2010, van Lutterveld et al., 2014). Howes, Shotbolt et al. (2013*) used PET imaging with [18F]-DOPA to investigate dopamine (DA) synthesis capacity in HVHs and HCs. No significant differences were found in whole striatal DA synthesis capacity or associative, limbic and sensorimotor functional subdivisions. Thus, the dopaminergic dysregulation observed in psychosis (Howes, Williams et al., 2013) appears not to be present in HVHs. Similarly, in a verbal fluency paradigm (Diederen et al., 2010*), HVHs and HCs did not differ significantly on language lateralisation. CVHs showed greater activation in the right precentral gyrus and left insula than both HVHs and HCs. CVHs also showed greater activation in the right superior parietal lobule than HCs, who did not differ significantly from HVHs.

However, some neurobiological indices appear more similar in HVHs and CVHs. For instance, BOLD contrast fMRI during AVHs was not able to distinguish HVHs and CVHs (Diederen et al., 2012*). Furthermore, Diederen et al. (2013*) reported that during resting-state, HVHs exhibit aberrant connectivity of frontal, superior temporal and parahippocampal areas compared to HCs. Although no CVH sample was included, the authors point towards similar findings in clinical populations, and hypothesise that such alterations underlie the failure of inner speech to be attributed as self-generated. De Weijer et al. (2013*) used DTI and magnetization transfer imaging to compare integrity of white matter tracts in CVHs, HVHs and HCs. For the left arcuate fasiculus, both CVHs and HVHs had higher magnetisation transfer ratios than HCs, further suggesting some alterations in white matter connectivity, whilst only CVHs had higher magnetisation transfer ratios in the right arcuate fasiculus compared to HCs but not HVHs, who did not differ significantly from each other. Fractional anisotropy was significantly lower in left arcuate fasiculus, right cortico-spinal tract and bilateral uncinate fasiculi for CVHs only, suggesting altered connectivity and white matter abnormalities to be largely specific to CVHs.

Van Lutterveld et al. (2014*) conducted a structural MRI study, comparing CVHs, HVHs and HCs. There were significant group differences in left paracentral lobule, left pars orbitalis, right fusiform gyrus and right inferior temporal gyrus, with CVH lowest, HVH intermediate and HCs showing highest cortical thickness. Right insula thickness was decreased in both CVHs and HVHs compared to controls. In another study however, EEG measures of the oddball paradigm showed activation patterns consistent with increased effortful attention in HVHs, a finding diametrically opposed of that typically observed in psychosis patients (van Lutterveld et al., 2010*). The authors hypothesise that the oddball paradigm is therefore not associated with AVHs per se. Indeed, most of the studies found no association between the neuroimaging indices and assessed AVH parameters (e.g. frequency or emotional valence). This was the case for cortical thickness (van Lutterveld et al., 2014*), striatal dopamine synthesis (Howes et al., 2013, Howes et al., 2013), lateralization indices (Diederen et al., 2013*) as well as fractional anisotropy and magnetization transfer ratios (de Weijer et al., 2013*). Thus, with several of these measures it appears likely that the investigated parameter is not AVH-related, but population-specific.

### 3.10 Trauma exposure

All of the five studies that assessed trauma in HVHs reported increased rates of trauma exposure similar to those in CVHs. Honig et al. (1998) first found evidence of elevated trauma rates in HVHs: whilst childhood trauma rates were significantly higher in CVHs than HVHs, only 27% of HVHs had no history of childhood abuse. Unlike Honig et al., but in a much larger sample, Daalman et al. (2012*) found that CVHs and HVHs did not differ significantly from each other in prevalence of childhood sexual, physical or emotional abuse, or physical or emotional neglect, which were all higher than in HCs (Sommer, Daalman et al., 2010*). Type of trauma did not predict emotional valence or phenomenology of voices. Similarly, Andrew et al. (2008) found no significant differences in exposure rates to traumatic childhood or adulthood events between CVHs and HVHs, although CVHs had higher rates of childhood sexual abuse. Traumatic events were more closely associated with PTSD symptoms in CVHs than HVHs. Trauma predicted beliefs of high malevolence, low benevolence and high omnipotence of voices, as well as higher levels of anxiety. Kråkvik et al. (2015) found higher rates of lifetime trauma exposure in HVHs compared to HCs, but lower than in CVHs. CVHs and HVHs did not differ in their experience of bullying, although a trend-level effect was observed suggesting higher rates in CVHs, and both groups were significantly higher than HCs. Notably, the age of exposure was not assessed. A significantly larger percentage of CVHs had been in dangerous situations or accidents than HVHs, who in turn had a larger exposure to such events than HCs. Interestingly, HVHs were significantly less likely to identify such stressful life events as related to AVH onset, in contrast to CVHs.

### 3.11 Familial risk

Three of the identified studies reported on the potential familial risk of HVHs. In Linden et al. (2011), 2 of 7 HVH participants reported a first-degree relative with psychosis. Similarly, van Lutterveld et al. (2014*) reported that HVHs and CVHs had a greater number of first- and second-degree relatives with a psychotic disorder compared to HCs, and they did not differ between each other. Notably, no group differences in the number of relatives with a manic disorder were observed. Conversely, Sommer, Daalman et al. (2010*) reported that relatives of HVHs had significantly higher prevalence rates of depressive disorders, mania and substance use disorders than HCs, with a similar trend for psychosis, suggesting higher rates of mental illness in families of both HVHs and CVHs. Further, such apparent genetic loading was predictive of global functioning.

## 4 Discussion

This systematic review identified a total of 36 studies investigating HVHs, spanning various study designs from small qualitative to large epidemiological studies. The literature includes studies investigating voice phenomenology, their impact and appraisal, mood disturbances, impairment and functioning, related psychotic phenomena, cognitive functioning, neuroimaging, trauma exposure and familial risk. Sampling methodologies vary widely, with HVH sample sizes ranging from six to 183, and variable recruitment of HC and/or CVH control samples. The findings need to be interpreted in the context of a number of limitations in the existing literature, which are elaborated below. Most notably, 17 of the 36 reviewed studies are based on variations of the same cohort, which may skew results according to the sampling methodology of those studies, and may inflate the consistency of some of the findings.

### 4.1 Phenomenology and impact

Contrary to what would be predicted by diagnostic models, the phenomenology of AVHs is overall similar in HVH and CVHs, particularly in form (e.g. loudness or location), but less so in content and incidence (i.e., frequency and duration). However, the selection of samples based on minimum frequency scores of AVHs may lead to a distortion of the phenomenology of AVHs. Wider populations, where AVHs may be distributed with lower frequency, are excluded in most studies. Thus, it cannot be ruled out that parameters such as AVH loudness are actually attenuated once frequency decreases. Large epidemiological research focused on AVHs is necessary to describe such patterns more accurately, with study designs such as those employed by Woods et al. (2015) and Kråkvik et al. (2015).

The impact and appraisal of AVHs differ substantially between HVHs and CVHs, as would be predicted by a fully-dimensional model where AVHs themselves are insufficient to cause distress. Negative beliefs about voices, such as attributed malevolence and omnipotence, were often predictive of mood disturbances and negative emotional reactivity, as hypothesised by cognitive models of voices (Chadwick & Birchwood, 1994). CVHs consistently report diminished control over their voices, with diminished control as well as need for control being predictive of voice-distress. Although it is likely that the distress of clinical voice-hearers is driven by increased frequencies and negative voice content, a role of ‘top-down processes’ in driving phenomenological characteristics cannot be ruled out. For instance, resistant relationships with voices, a coping style predominantly employed by CVHs, may partially account for the increased frequencies and duration of AVHs in CVHs. HVHs reported that resistance led to initial distress, which was mitigated by engagement (i.e. acceptance and understanding) (Taylor & Murray, 2012). Indeed, HVHs are more likely to have a mindful response style to voices (Peters et al., 2016). This is reminiscent of the thought suppression literature, where it has been found that actively trying to suppress thoughts paradoxically increases their repetitiveness and intrusiveness (Wenzlaff & Wegner, 2000). Thus, it should not be ruled out that phenomenology of voices is shaped by their interactions with ‘top-down’ processes such as appraisals and coping strategies. Similarly, the negative content of voices may be shaped by the presence of mood difficulties, distress or low self-esteem in CVHs, as suggested by the evidence on mood-congruent AVHs (Larøi, 2012). In turn, the well-replicated finding that AVH onset occurs significantly earlier in HVHs may explain divergent cognitive appraisals. It could be speculated that earlier onset can be protective against negative appraisals such as thinking that one is “crazy”, as societal stigmatising implications of AVHs may not be understood at that age. However, in the absence of consistent epidemiological and longitudinal evidence, the cross-sectional evidence reported in the literature makes it difficult to determine the direction of relationships amongst AVHs variables and outcomes.

### 4.2 Mental health and functioning

Greater rates of depression and anxiety are reported in CVHs compared to HVHs. The relative lack of mood disorders in HVHs again does not support a diagnostic or quasi-dimensional framework, i.e. persistent AVHs can occur independent of distress and mood disturbances. However, these findings need to be viewed in the context of sample selection and stratification in most studies, most notably those of the Utrecht cohort (see Table 2) which applied very strict eligibility criteria (i.e., exclusion of any current psychiatric disorder or substance use). Indeed, Kråkvik et al. (2015), using a more open, epidemiological design, did find higher rates of mental health problems in HVHs compared to HCs. Daalman et al. (2016*) further showed that despite good mental health at baseline, their HVHs were at higher risk of developing a need for mental healthcare, most strongly predicted by voice distress and previous mood disturbances. Most of the studies investigating global functioning also showed increased levels of impairments in HVHs compared with HCs, although these tended to be of subclinical magnitude and situated on a continuum between HCs and CVHs. The reviewed studies thus suggest that although HVHs mostly do not require care and suffer no distress (a finding in line with the fully-dimensional model), there is nonetheless some evidence of an increased risk of need for care from epidemiological or longitudinal research (a finding in line with the quasi-dimensional model). Similarly, HVHs score higher than HCs, but lower than CVHs, on disorganization of thought, show higher levels of delusional ideation than HCs, and have more implicit cognitive biases than HCs, but less than CVHs. Interpreted from a multidimensional standpoint, this may imply that mood disorder and distress are only weakly associated with AVHs, which in turn are more consistently associated with other positive symptoms and cognitive biases. However, since much of the evidence is cross-sectional, it is as of yet impossible to disentangle causal pathways.

The strictly dichotomous stratification in the majority of studies means that clinical individuals who are in remission, or generally healthy individuals who show occasional, subclinical distress, are often excluded in research. Given that the present literature was born out of a reconceptualization of psychosis towards dimensional models, it is paradoxical that the gray zone in which transitions to and from care-necessitating disturbances occur remains largely unexplored. Differing psychological factors in HVHs and CVHs such as cognitive biases or voice appraisals can, and already do, inform cognitive-behavioural interventions. Therefore, cross-sectional as well as longitudinal research of such transitioning populations, such as that carried out in the literature on at-risk populations, may be most relevant to clinical care and should be addressed in future research.

### 4.3 Risk factors

HVHs consistently report the presence of well-established latent risk factors for psychosis, i.e. genetic loading (Howes et al., 2016) and childhood trauma (Varese et al., 2012). Whilst a greater degree of risk exposure would be expected for both groups in Models 2 & 3, it is striking that HVHs and CVHs show almost no difference in exposure to these specific risk factors. However, whilst familial incidence of psychiatric disturbances is a reasonable indicator of genetic risk, heritability estimates of AVHs in CVHs and HVHs, as well as molecular genetic and epigenetic investigations, are needed for a more comprehensive understanding. Additionally, a strong case is made for the role of childhood trauma, which was consistently elevated in HVHs across all studies, a finding in line with the highly predictive impact of childhood trauma in the emergence of AVHs demonstrated in other studies (Read et al., 2005, Shevlin et al., 2008). This high rate of trauma exposure in HVHs may also explain the greater risk for distress in HVHs compared to non-voice-hearing members of the general population. Future research should address whether trauma exposure underlies the association of AVHs and distress in the general population.

However, variables such as socioeconomic status or positive social relationships, which may act as further risk or protective factors, have remained unexplored in this context despite their potential relevance. Indeed, in the context of wider psychotic experiences, Peters et al. (2016) showed that non-clinical individuals were less likely to be members of a minority ethnic group, come from a working class background, live in areas with civic disorder, and were more likely to be employed, have higher educational achievements, and have meaningful relationships. Future research should further investigate adulthood exposure to adversity, stressful life events and everyday stress to assess whether CVHs have greater exposure to the “third hit” proposed in 3-hit models of stress vulnerability (Daskalakis, Bagot, Parker, Vinkers, & de Kloet, 2013). That is, if HVHs and CVHs largely share the first hit, i.e., a genetic susceptibility, and the second hit, i.e., exposure to childhood traumatic events, then a third hit, i.e., in the form of adversity exposure in early adulthood, may crucially shape the clinical trajectory. The age of exposure to trauma is of great importance for such an assessment and has been omitted in all of the identified studies. Of note, it is surprising that the role of drug use as a risk factor has not been assessed in the literature, potentially due to stringent sampling procedures. However, evidence by Peters et al. (2016) suggests that non-clinical individuals who report wider psychotic experiences are less likely to use drugs than both their clinical counterparts and HCs, a finding that needs replication specifically in the context of HVHs.

### 4.4 Neurobiology

Several neuro-cognitive and biological variables appear inconclusive in regards to the three frameworks, at least in some domains. Whilst findings on cortical thickness (van Lutterveld et al., 2014*) and white-matter integrity (de Weijer et al., 2013*) are broadly in line with quasi- and fully-dimensional models, several of the functional paradigms showed incongruences with such models. Notably, language lateralisation does not differ between HVHs and HCs, but differs from CVHs (Diederen et al., 2013, Diederen et al., 2010). EEG-measured response to the auditory oddball paradigm in HVHs diverges from HC populations indicating increased effortful attention, directionally opposing the well-replicated finding that psychosis is associated with decreased effortful attention (van Lutterveld et al., 2010*). The authors suggest that this primarily indicates that AVHs are unrelated to effortful attention, as correlations of reduced P300 amplitudes with positive or negative symptoms in schizophrenia patients have not been consistently replicated. Notably, this issue translates to several of the investigated variables: it is often difficult to disentangle whether a particular finding is a substrate of AVHs, or a byproduct of wider symptomatology and population differences. Thus, for many of these findings it is not clear whether apparent discontinuity is ultimately one of the phenomenon or the population.

Interestingly, Howes, Shotbolt, et al. (2013*) and Howes, Williams et al. (2013*) reported no differences in DA synthesis capacity between HVHs and HCs. Increased striatal DA synthesis capacity has been a consistent finding in psychosis patients (Fusar-Poli and Meyer-Lindenberg, 2013, Howes et al., 2012) and has also been reported in at-risk individuals (Howes et al., 2011). According to the DA hypothesis (Howes & Kapur, 2009), increased striatal DA signaling leads to aberrant salience attribution to unwarranted stimuli and their associations. Whilst this is hypothesised to lead to the formation of delusional explanations, it is not established whether DA dysregulation actually underlies hallucinatory experiences. The authors (Howes et al., 2013, Howes et al., 2013) conclude that their findings suggest that, at least in the case of non-clinical AVHs, this is not likely to be the case. Speculatively, dysregulated DA synthesis may act as a moderating factor upon which the formation of delusional beliefs secondary to AVHs is contingent, such as threatening appraisals. However, when variables directly associated with AVHs are considered, CVHs and HVHs appear highly similar; for instance, Diederen et al. (2012*) found no differences between CVHs and HVHs in brain activity during acute AVHs, suggesting a shared neurobiological mechanism underlying AVHs in both groups.

### 4.5 Conclusions

The evidence considered in the present systematic review does not support strictly categorical or disease models of psychotic experiences, and is generally inconsistent with a diagnostic conceptualization (Model 1), thus supporting the first hypothesis. Instead, the evidence supports fully-dimensional and quasi-dimensional models (Models 2 and 3) to a similar degree, and cannot distinguish between them, as predicted by the second hypothesis. Therefore a hybrid conceptualization is likely to be the most accurate model (Linscott and van Os, 2013, van Os and Reininghaus, 2016). Characteristics of individual symptoms (e.g. frequency, loudness or content of AVHs) may differ in their continuity between populations, and may feasibly present with skewed or bimodal rather than normal distributions. Especially in the case of bimodal distribution, the contrast between continuous and categorical is left as a primarily semantic issue, as even the most categorical distinctions (e.g. gender) have blurry boundaries (e.g. hermaphroditism or non-binary gender identities). Thus, whilst the evidence ultimately suggests continuity, it is upon future epidemiological research to tease out the complexities and relationships of symptom dimensions. Nonetheless, research on AVHs in healthy populations may prove of crucial value to the understanding and treatment of AVHs in clinical populations.

## Figures and Tables

**Table 1:**
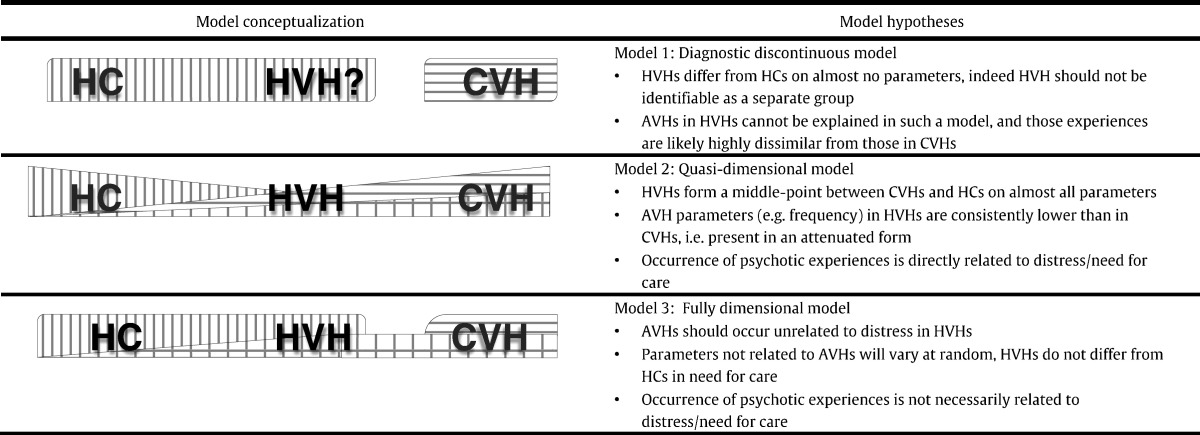
Model conceptualisations and hypotheses. Vertical shading indicates mental well-being or the absence of need for care, horizontal shading indicates psychological difficulties and need for care, and grid shading indicates the occurrence (e.g., frequency, intensity) of psychotic experiences.

**Table 2:** Systematic overview of individual studies, their recruitment strategy, selection criteria, sample age, sample gender proportions (in % female), and study measures; CVH – Clinical Voice Hearer; HVH – Healthy Voice Hearers; HC – Healthy Control; Gen Pop – General Population Sample; BPD – Borderline Personality Disorder; SZ – Schizophrenia; CNVH – Clinical participants who do not hear voices; C – Christian; NR – Non-religious; PE – Healthy individuals with psychotic experiences; CPE – Clinical individuals with psychotic experiences; n/a – not available; * - indicates that studies belong to the same Dutch cohort; ^1^ – no separate means provided.

Study	Sample	Group characteristics	AVH Selection	Mean Age	Gender (% female)	Measures
1. Andrew et al., 2008	22 CVHs 21 HVHs	CVHs were recruited from mental health services, HVHs were recruited from spiritualist sources. Psychiatric status in HVHs was not formally assessed and meeting criteria for a psychiatric diagnosis was not amongst the exclusion criteria. Anyone with an organic condition that may cause AVHs was excluded	Participants were self-selcted for experiencing “clairaudience”. Presence of AVHs was assessed via PSYRATS but was not a formal part of selection procedures.	CVHs: 39.6 HVHs: 50.7	CVHs: 40.9 HVHs: 71.4	- Psychotic Symptoms Rating Scale Auditory Hallucinations Subscale - Beliefs About Voices Questionnaire – Revised - Post-traumatic Diagnostic Scale - Impact of Events Scale - Beck Anxiety Inventory - Beck Depression Inventory - II
2. Beavan & Read, 2010	84 CVH 69 HVH (collapsed sample)	Clinical status was assigned by stratifying for mental health service contact.	Self-selected individuals who responded to having “heard voices that no one else can hear”.	48.0^1^	66.0^1^	- Hearing Voices Questionnaire - Qualitative interview
3. Begemann et al., 2016*	101 HVH 101 HC	As Daalman, Boks et al., 2011a	As Daalman, Books et al., 2011a	n/a	n/a	- Stroop Color-Word Task - WAIS-III - Childhood Trauma Questionnaire
4. Cottam et al., 2011	15C-CVH 14 NR-CVH 20C-HVH	HVH-Cs were recruited from churches. They were not formally assessed for psychiatric status. CVHs were recruited from mental health services.	Participants were included if they endorsed the LSHS item “In the past I have had the experience of hearing a voice and then found no one was there”.	CVH-C: 41.8 CVH-NR: 41.0 HVH-C: 52.7	CVH-C: 20.0 CVH-NR: 21.0 HVH-C: 60.0	- Launay-Slade Hallucination Scale - Topography of Voices Rating Scale - Affective Experiences Questionnaire - Cognitive Assessment of Auditory Hallucinations (supplemented with questions for religious belief and interpretation)
5. Daalman, Boks et al., 2011*	118 CVH 111 HVH	HVHs were excluded if they met criteria for a DSM-IV diagnosis other than depressive or anxiety disorders in complete remission. Individuals were screened for illegal substance use via urine samples, and alcohol or drug abuse in the last 3 months led to exclusion. HVHs were recruited online, CVHs were recruited from mental health services. CVHs consisted of patients with schizophrenia, schizoaffective disorder and psychosis not otherwise specified.	Participants were initially screened with LSHS items concerning having heard a person's voice when no-one was there and having been troubled by voices in their head. Voices had to be distinct from thoughts and have a perceptual quality, minimum frequency for AVHs in HVHs was once per month and minimum duration since onset was 1 year.	CVH: 36.6 HVH: 41.5	CVH: 40.0 HVH: 71.0	- Psychotic Symptoms Rating Scale Auditory Hallucinations Subscale - Structured Clinical Interview for DSM-IV II - Personality Disorders - Comprehensive Assessment of Symptoms and History
6. Daalman, van Zandvoort et al., 2011*	101 HVH 101 HC	As in Daalman, Boks et al. 2011. All participants had an IQ of 80 or above.	As Daalman, Boks et al., 2011, except minimum frequency of AVH was once every 3 months for at least 1 year.	HVH: 43.8 HC: 43.3	HVH: 66.3 HC: 70.3	- Psychotic Symptoms Rating Scale Auditory Hallucinations Subscale - Stroop Color-Word Task - Wechsler Adulthood Intelligence Scale III subtasks (backward digit span-task, forward digit span-task, vocabulary test, similarities test) - California Verbal Learning Test - Complex Figure of Rey-Osterrieth - Controlled Oral Word Association Test - Semantic Fluency Test - National Adult Reading Test - Raven's Advanced Progressive Matrices - Structured Clinical Interview for DSM-IV II - Personality Disorders - Comprehensive Assessment of Symptoms and History
7. Daalman, Diederen et al., 2012*	40 CVH 40 HVH 40 HC	As Daalman, Boks et al., 2011	As Daalman, Boks et al., 2011	CVH: 37.6 HVH: 47.6 HC: 45.0	CVH: 47.5 HVH: 60.0 HC: 55.0	- Psychotic Symptoms Rating Scale Auditory Hallucinations Subscale - Launay-Slade Hallucination Scale - Semantic Expectation Task - Structured Clinical Interview for DSM-IV II - Personality Disorders - Comprehensive Assessment of Symptoms and History
8. Daalman et al., 2012*	100 CVH 127 HVH 124 HC	As Daalman, Boks et al., 2011	As Daalman, Boks et al., 2011	CVH: 38.0 HVH: 42.4 HC: 43.1	CVH: 56.0 HVH: 67.7 HC: 67.7	- Psychotic Symptoms Rating Scale Auditory Hallucinations Subscale - Childhood Trauma Questionnaire - Structured Clinical Interview for DSM-IV II - Personality Disorders - Comprehensive Assessment of Symptoms and History
9. Daalman et al., 2013*	72 CVH 72 HVH 72 HC	As Daalman, Boks et al., 2011	As Daalman, Boks et al., 2011, except minimum frequency of AVH was once every 3 months for at least1 year.	CVH: 39.7 HVH: 47.6 HC: 45.1	CVH: 54.2 HVH: 69.4 HC: 72.2	- Psychotic Symptoms Rating Scale Auditory Hallucinations Subscale - Cognitive Biases Questionnaire for Psychosis - Structured Clinical Interview for DSM-IV II - Personality Disorders - Comprehensive Assessment of Symptoms and History
10. Daalman et al., 2016*	81 HVH 49 HC	As Daalman, Boks et al., 2011; 5-year follow-up (thus healthy status may not apply)	As Daalman, Boks et al., 2011	n/a	n/a	- Psychotic Symptoms Rating Scale Auditory Hallucinations Subscale - Comprehensive Assessment of Symptoms and History
11. Davies et al., 2001	18 CVH 17C-HVH 12C-HC 15 NR-HVH 40 NR-HC	Evangelical groups reported being born-again Christians or members of evangelical Christian churches, and reported no previous treatment for mental illness. No evangelical Christians were in the CVH group. All CVHs had a diagnosis of schizophrenia.	Participants were included in voice-hearer groups if they endorsed the LSHS item “In the past I have had the experience of hearing a voice and then found no one was there”.	CVH: 32.6 E: 33.3 NR: 33.0	CVH: 61.1 E: 69.0 NR: 63.6	- Launay-Slade Hallucination Scale - Affective Experiences Questionnaire - Perceptions of Voices Questionnaire
12. De Weijer et al., 2013*	35 CVH 35 HVH 36 HC	As Daalman, Boks et al., 2011 CVH group reported AVH at least once an hour.	As Daalman, Boks et al., 2011	CVH: 39.6 HVH: 42.1 HC: 41.4	CVH: 60.0 HVH: 62.9 HC: 61.1	- Psychotic Symptoms Rating Scale Auditory Hallucinations Subscale (items for frequency, emotional valence, distress and control) - Positive and Negative Syndrome Scale - Global Assessment of Functioning Scale - Schizotypal Personality Questionnaire (HVH & HC only) - Diffusion Tensor Imaging - Magnetisation Transfer Imaging - Comprehensive Assessment of Symptoms and History - Edinburgh Handedness Inventory
13. Diederen et al., 2010*	35 CVH 35 HVH 35 HC	As Daalman, Boks et al., 2011	As Daalman, Boks et al., 2011	CVH: 43.6 HVH: 44.3 HC: 41.7	CVH: 68.6 HVH: 68.6 HC: 65.7	- Psychotic Symptoms Rating Scale Auditory Hallucinations Subscale - Positive and Negative Syndrome Scale - Global Assessment of Functioning Scale - BOLD fMRI during paced verbal fluency task - Structured Clinical Interview for DSM-IV II - Personality Disorders - Comprehensive Assessment of Symptoms and History - Schizotypal Personality Questionnaire
14. Diederen et al., 2012*	21 CVH 21 HVH	As Daalman, Boks et al., 2011	As Daalman, Boks et al., 2011	CVH: 34.0 HVH: 46.5	CVH: 81.0 HVH: 76.2	- Psychotic Symptoms Rating Scale Auditory Hallucinations Subscale - Positive and Negative Syndrome Scale - Global Assessment of Functioning Scale - Schizotypal Personality Questionnaire - BOLD fMRI during AVHs (indicated by balloon squeezes) - Structured Clinical Interview for DSM-IV II - Personality Disorders - Comprehensive Assessment of Symptoms and History
15. Diederen et al., 2013*	25 HVH 25 HC	As Daalman, Boks et al., 2011	As Daalman, Boks et al., 2011a	HVH: 41.6 HC: 39.8	HVH: 72.0 HC: 72.0	- Global Assessment of Functioning Scale - Schizotypal Personality Questionnaire - BOLD fMRI during resting-state - Structured Clinical Interview for DSM-IV II - Personality Disorders - Comprehensive Assessment of Symptoms and History
16. Fleming & Martin, 2009	19 HVH 102 HC	Mental health practitioners.	Convenience sample was assessed on the prevalence of psychotic symptoms using the PSYRATS	–	–	- Hospital Anxiety and Depression Scale - Psychotic Symptoms Rating Scale
17. Hill et al., 2012	20 CVH 20 HVH 20 HC	HVHs were recruited from spiritualist sources and opportunity sampling, CVHs were recruited from mental health services, HCs were recruited via opportunity sampling. None of the HVHs or HCs had a psychiatric diagnosis or were receiving treatment.	Presence of AVHs was assessed via PSYRATS.	CVH: 36.2 HVH: 39.2 HC: 37.4	CVH: 65.0 HVH: 60.0 HC: 50.0	- Positive and Negative Syndrome Scale - Psychotic Symptoms Rating Scale Auditory Hallucinations Subscale - Meta-Cognitive Questionnaire (Short Version)
18. Honig et al., 1998	33 CVH 15 HVH	HVHs were included if they had no previous psychiatric history. CVHs were recruited from mental health services, HVH were recruited via opportunity sampling and voice-hearer groups.	CVHs had to have persistent AVHs over the last 6 months, HVHs not specified.	CVH: 38.4 HC: 56.0	CVH: 75.6 HC: 73.0	- Semi-structured interview covering characteristics of voices, history of voices, triggers, interpretations of voices, coping strategies and traumatic life events - Dissociative Experience Scale - Composite International Diagnostic Interview
19. Howes et al., 2013, Howes et al., 2013	16 HVH 16 HC	As Daalman et al., 2011a	As Daalman et al., 2011a	HVH: 43.9 HC: 42.8	HVH: 68.8 HC: 62.5	- Peters Delusion Inventory - Psychotic Symptoms Rating Scale - Schizotypal Personality Questionnaire - Global Assessment of Functioning Scale - [18F]-DOPA Positron Emission Tomography - Structured Clinical Interview for DSM-IV II - Personality Disorders - Comprehensive Assessment of Symptoms and History
20. Jacobsen et al., submitted	39 CVH 35 HVH 77 HC	CVHs were recruited from two UK sites; HVHs were recruited as part of the wider UNIQUE study (see Peters et al., 2016).	Voice-hearers reported at least occasional voices on the Scale for the Assessment of Positive Symptoms	CVH: 41 HVH: 45 HCs: 45	CVH: 36 HVH: 74 HCs: 69	- Autobiographical Memory Task - Appraisals of Anomalous Experiences interview - Scale for the Assessment of Positive Symptoms - WAIS-III - BDI-II
21. Kråkvik et al., 2015	30 CVH 140 HVH 2359 HC	Swedish population cohort; 8000 contacted of whom 2533 responded with questionnaire data	Two LSHS items were used: “In the past I have had the experience of hearing a person's voice and then found that there was no-one there”and “I often hear a voice speaking my thoughts aloud”. Those who answered yes to both items were asked additional questions about voice characteristics.	CVH: 44.3 HVH: 42.2 HCs: 52.1	CVH: 43.3 HVH: 62.9 HCs: 54.3	- Launay-Slade Hallucination Scale - Hospital Anxiety and Depression Scale - Additional questions regarding stressful life events, voice phenomenology and mental health problems
22. Kompus et al., 2013	8 CVH 8 HVH 8 HC	HVHs were recruited via opportunity sampling. Individuals with sleep-related hallucinations were excluded, so were those with psychiatric treatment.	HVHs were characterised by either hearing voices when no one is around, or hearing their own thoughts as voices.	CVH: 31.1 HVH: 39.3 HC: 36.3	CVH: 62.5- HVH: 62.5 HC: 87.5	- Psychotic Symptoms Rating Scale - fMRI - Consonant-vowel Dichotic Listening Task - Hughson-Westlake Audiometric Test
23. Lawrence et al., 2010	184 HVH 71 CVH (external sample)	HVH individuals were only included if they had not sought psychiatric help for their voices, or heard voices when under the influence of substances.	Individuals were included if they reported currently hearing voices or having heard voices in the past.	HVH: 34.5 CVH: -	HVH: 68.5 CVH: -	- Hospital Anxiety and Depression Scale - Beliefs about Voices Questionnaire – Revised - Topography of Voices Rating Scale (3 items only)
24. Leudar et al., 1997	14 CVH 14 HVH	None of the HVHs were in contact with psychiatric services. Formal screening for psychiatric symptoms was not possible. Most HVHs were occasional cannabis users. CVHs were schizophrenia patients recruited from mental health services.	HVHs reported hearing voices in an on-going survey.	CVH: 31.7 HVH: 22.9	CVH: 35.71 HVH: 57.14	- Structured Interviews
25. Linden et al., 2011	7 HVH 7 HCs	Participants had no history of psychiatric or neurological illness and were recruited via opportunity sampling.	Recruitment occurred via self-reports, AVHs were assessed with PANSS.	HVH:45.0 HCs: 31.0	HVH: 71.4 HCs: 71.4	- Positive and Negative Syndrome Scale - Beliefs about Voices Questionnaire - BOLD fMRI during AVHs (indicated by button press)
26. Moritz & Larøi, 2008	46 CVH 17 HVH 69 CNVH 38 HC (partially collapsed samples)	Of the CVHs and CNVHs, 45 had schizophrenia, 60 had OCD. Individuals were classified as HCs and HVHs if they denied the presence of any psychiatric illness and contact with any mental health services.	AVHs were assessed as having heard voices when no-one was around.	GP: 35.6 OCD: 32.7 SZ: 35.9	GP: 65.5 OCD: 61.7 SZ: 46.7	- Yale-Brown Obessive Compulsive Scale - Community Assessment of Psychic Experiences - Interview assessment - Launay-Slade Hallucination Scale
27. Peters et al., 2016	92 PE 84 CPE 83 HC	Study recruited healthy individuals with psychotic experiences (PEs), who were assessed as having no need for care and no previous diagnosis for a psychotic disorder.	Voice-hearing was assessed using the Scale for the Assessment of Positive Symptoms. Analysis on voice-hearing was carried out only in relation to Southampton Mindfulness Questionnaire.	PE: 46 CPE: 42 HC: 46	PE: 72.8 CPE: 34.5 HC: 68.7	- Scale for the Assessment of Positive Symptoms - Southampton Mindfulness Questionnaire
28. Slotema et al., 2012*	38 BPD-CVH 51 SZ-CVH 66 HVH	As Daalman et al., 2011a	As Daalman et al., 2011a	BPD-CVH: 34.0 SZ-CVH: 37.0 HVH: 37.0	BPD-CVH: 100.0 SZ-CVH: 100.0 HVH: 100.0	- Structured Clinical Interview for DSM-IV II - personality disorders - Comprehensive Assessment of Symptoms and History - Psychotic Symptoms Rating Scale - Auditory Hallucinations
29. Sommer, Daalman et al., 2010*	103 HVH 60 HC	As Daalman et al., 2011a	As Daalman et al., 2011a	HVH: 44.0 HC: 46.0	HVH: 70.8 HC: 70.0	- Comprehensive Assessment of Symptoms and History - Psychotic Symptoms Rating Scale - Auditory Hallucinations - Launay-Slade Hallucination Scale - Global Assessment of Functioning Scale - Structured Clinical Interview for DSM-IV II - personality disorders - Schizotypal Personality Questionnaire - Peters Delusion Inventory - Revised NEO Personality Inventory - Childhood Trauma Questionnaire
30. Sommer, Derwort et al., 2010*	40 CVH 40 HVH 50 HC	As Daalman et al., 2011a	As Daalman et al., 2011a	CVH: 40.0 HVH: 41.0 HC: 44.0	CVH: 48.0 HVH: 62.0 HC: 70.0	- Comprehensive Assessment of Symptoms and History - Launay-Slade Hallucination Scale - Structured Clinical Interview for DSM-IV II - personality disorders - Schizotypal Personality Questionnaire - Peters Delusion Inventory - Thought and Language Index - Thematic Apperception Test - Global Assessment of Functioning Scale
31. Sorrell et al., 2010	32 CVH 18 HVH	Participants were excluded if they heard voices due to an organic illness or substance misuse. CVHs were recruited from mental health services. HVHs were excluded if they currently had contact with mental health services in relation to voice-hearing.	Individuals heard voices for at least 6 months.	CVH: 38.1 HVH: 54.3	CVH: 41.0 HVH: 67.0	- Psychotic Symptoms Rating Scale - Voice and You Questionnaire - Beliefs about Voices Questionnaire – Revised - Beck-Depression Inventory - II
32. Taylor & Murray, 2012	6 HVH	Participants were included if they reported no frequent distress and reported no contact with mental health services in relation to their voice experiences. Participants were self-identified mediums.	Self-reported “clairaudience” or hearing the voices of spirits was taken as a proxy for AVHs.	48.5	66.7	- Qualitative Interviews
33. Van Lutterveld et al., 2010*	18 HVH 18 HC	As Daalman et al., 2011a	As Daalman et al., 2011a	HVH: 42.8 HC: 43.8	HVH: 83.3 HC: 83.3	- Comprehensive Assessment of Symptoms and History - Structured Clinical Interview for DSM-IV II - personality disorders - Schizotypal Personality Questionnaire - Peters Delusion Inventory - EEG - auditory oddball paradigm
34. Van Lutterveld et al., 2014*	50 CVH 50 HVH 50 HC	As Daalman et al., 2011	As Daalman, Bolts et al., 2011	CVH: 39.9 HVH: 40.8 HC: 40.5	CVH: 62.0 HVH: 62.0 HC: 62.0	- Comprehensive Assessment of Symptoms and History - Structured Clinical Interview for DSM-IV II - personality disorders - Positive and Negative Syndrome Scale - Schizotypal Personality Questionnaire - Global Assessment of Functioning Scale - MRI
35. Varese et al., 2016	18 HVH 22 CVH	Recruitment sources not clear	HVHs were individuals with no current or past mental health difficulties. CVHs had previously received diagnoses.	CVH: 37.8 HVHs: 39.9	CVH: 45.5 HVH: 38.9	- Psychotic Symptoms Rating Scale - Cognitive Assessment of Voices Interview - Modified Goals Task
36. Woods et al., 2015	26 HVH 127 CVH	Online questionnaire; anyone hearing voices was free to participate. Some subgroup analyses were carried out in relation to whether individuals had received mental health care.	Participants were included if they reported hearing voices.	n/a; range 16–84	65.4	- Self-reported qualitative questionnaire comprising 13 items

**Table 3:** Voice phenomenology findings; **↑** indicates greater in HVHs than CVHs, ↓ indicates lower in HVHs than CVHs, = indicates similar in HVHs and CVHs, − indicates that no results were available for the parameter; * indicates that studies belong to the same Dutch cohort; † when comparing HVHs to CVHs with a diagnosis of schizophrenia (as opposed to OCD).

Study	Duration	Frequency	Loudness	Location	Beliefs of Origin	Number of Voices	Negative Voice Content/Valence	Control	Disruption
Andrew et al., 2008	↓	↓	↓	=	–	–	↓	↑	–
Cottam et al., 2011	–	↓	↓	–	–	–	–	–	–
Daalman, Boks et al., 2011*	↓	↓	=	=	↑ external	↓ 3rd person	↓	↑	–
Daalman et al., 2012*	↓	↓	=	=	↑ external	–	↓	↑	–
Daalman, Verkooijen et al., 2012*	↓	↓	=	–	↑ external	–	↓	↑	–
Daalman et al., 2013*	↓	↓	=	=	↑ external	–	↓	↑	↓
Davies et al., 2001	–	↓	–	–	–	–	–	–	–
de Weijer et al., 2013*	**–**	↓	–	–	–	–	↓	**↑**	–
Diederen et al., 2010*	↓	↓	=	=	↑ external	↓	↓	**↑**	–
Diederen et al., 2012*	= during scanning	↓	=	=	=	=	↓	**↑**	–
Hill et al., 2012*	=	=	=	=	=	=	↓	=	↓
Honig et al., 1998	↓	↓	–	=	–	↓ 3rd person	↓	**↑**	↓
Kråkvik et al., 2015	–	↓	–	–	–	–	↓	–	↓
Leudar et al., 1997	–	–	**–**	↑ inside head	–	–	↓	–	–
Moritz & Larøi, 2008^†^	–	–	**↑**	**–**	–	–	**–**	=	↓
Slotema et al., 2012*	↓	↓	=	=	**↑** external	–	↓	**↑**	↓
Sommer, Derwort et al., 2010*	**–**	↓	**–**	**–**	**–**	–	**–**	–	**–**
Sorrell et al., 2010	**–**	**–**	**–**	**–**	**–**	–	↓	–	**–**

**Table 4:** Results of neuroimaging studies; ↑ indicates an increase, ↓ indicates a decrease, = indicates no difference, n/a indicates that no such comparison was conducted.

Study	Paradigm	Tested association		
		HVH compared to CVH	HVH compared to HC	CVH compared to HC
De Weijer et al., 2013*	Diffusion Tensor Imaging Magnetisation Transfer Imaging	↑ Fractional anisotropy in left arcuate fasiculus, right cortico-spinal tract and bilateral uncinate fasiculi	↑ Magnetisation transfer ratio in left arcuate fasiculus	↑ Magnetisation transfer ratio in left arcuate fasiculus ↑ Magnetisation transfer ratio in right arcuate fasiculus ↑ Radial diffusivity in the right arcuate fasicilus
Diederen et al., 2010*	BOLD fMRI during verbal fluency task	↑ Lateralisation ↓ Activation in right precentral gyrus and left insula	–	↓ Lateralisation ↑ Activation in right precentral gyrus, left insula, and right superior parietal lobule
Diederen et al., 2012*	BOLD fMRI during AVHs	= Activation in a priori hypothesised regions, comprising bilaterial inferior frontal gyri, insula, superior and middle temporal gyri, supramarginal gury, precentral and post-central gyri, cerebellum, hippocampus and parahippocampal gyrus, as well as across all gray matter voxels = Lateralisation indices	n/a	n/a
Diederen et al., 2013*	BOLD fMRI during resting-state	n/a	↓ Connectivity of left superior temporal gyrus with right and left superior temporal regions ↑ Connectivity of left parahippocampal gyrus with left inferior frontal region = Connectivity of right superior temporal and bilateral inferior frontal regions No negative correlation of right inferior frontal gyrus activity with left temporoparietal region in HVHs	n/a
Howes et al., 2013, Howes et al., 2013	[18F]-DOPA Positron Emission Tomography	n/a	= Whole striatal dopamine synthesis capacity as well as in associative, limbic and sensorimotor functional subdivisions	n/a
				
Kompus et al., 2013	fMRI during dichotic listening task	n/a	↓ Primary auditory cortex activation in response to stimulation	n/a
Linden et al., 2011	BOLD fMRI during AVHs (vs imagined voices in HCs)	n/a	↑ Activation in bilateral inferior parietal lobules, left middle frontal gyrus, posterior cingulate cortex, left Heschl's gyrus and bilateral calcarine sulci ↑ Time of onset of activity in supplementary motor area, followed by bilateral inferior frontal gyri and superior temporal sulcus	n/a
Van Lutterveld et al., 2010*	EEG during oddball paradigm	n/a	↑ P300 amplitudes, processing negativity amplitudes = P300 latency, processing negativity latency, mismatch negativity amplitude and and latency	n/a
Van Lutterveld et al., 2014*	MRI	↑ Cortical thickness in left paracentral lobule, left pars orbitalis, right fusiform gyrus and right inferior temporal gyrus	↓ Cortical thickness in left paracentral lobule, left pars orbitalis, right fusiform gyrus and right inferior temporal gyrus ↓ Right insula thickness	↓ Cortical thickness in left paracentral lobule, left pars orbitalis, right fusiform gyrus and right inferior temporal gyrus ↓ Right insula thickness

## References

[bb0005] Andrew E.M., Gray N.S., Snowden R.J. (2008). The relationship between trauma and beliefs about hearing voices: A study of psychiatric and non-psychiatric voice hearers. Psychological Medicine.

[bb0010] Badcock J.C., Chhabra S. (2013). Voices to reckon with: Perceptions of voice identity in clinical and non-clinical voice hearers. Frontiers in Human Neuroscience.

[bb0015] Badcock J.C., Hugdahl K. (2012). Cognitive mechanisms of auditory verbal hallucinations in psychotic and non-psychotic groups. Neuroscience and Biobehavioral Reviews.

[bb0020] Beavan V., Read J. (2010). Hearing voices and listening to what they say: The importance of voice content in understanding and working with distressing voices. The Journal of Nervous and Mental Disease.

[bb0025] Begemann M.J.H., Daalman K., Heringa S.M., Schutte M.J.L., Sommer I.E.C. (2016). Letter to the Editor: Childhood trauma as a risk factor for psychosis: The confounding role of cognitive functioning. Psychological Medicine.

[bb9000] Bell V., Halligan P.W., Ellis H.D. (2008). Are anomalous perceptual experiences necessary for delusions?. The Journal of nervous and mental disease.

[bb0030] Bentall R.P. (2003). Madness Explained: Psychosis and Human Nature London.

[bb0040] Chadwick P., Birchwood M. (1994). The omnipotence of voices. British Journal of Psychiatry.

[bb0045] Cottam S., Paul S.N., Doughty O.J., Carpenter L., Al-Mousawi A., Karvounis S., Done D.J. (2011). Does religious belief enable positive interpretation of auditory hallucinations?: A comparison of religious voice hearers with and without psychosis. Cognitive Neuropsychiatry.

[bb9005] Claridge G. (1994). Single indicator of risk for schizophrenia: Probable fact or likely myth?. Schizophrenia Bulletin.

[bb9010] Claridge G., Beech T., Raine A., Lencz T., Mednick S.A. (1995). Fully and quasi[HYPHEN]dimensional constructions of schizotypy. Schizotypal personality.

[bb0050] Daalman K., Boks M.P.M., Diederen K.M.J., De Weijer A.D., Blom J.D., Kahn R.S., Sommer I.E.C. (2011). The same or different? A phenomenological comparison of auditory verbal hallucinations in healthy and psychotic individuals. Journal of Clinical Psychiatry.

[bb0055] Daalman K., van Zandvoort M., Bootsman F., Boks M., Kahn R., Sommer I. (2011). Auditory verbal hallucinations and cognitive functioning in healthy individuals. Schizophrenia Research.

[bb0060] Daalman K., Diederen K.M.J., Derks E.M., van Lutterveld R., Kahn R.S., Sommer I.E.C. (2012). Childhood trauma and auditory verbal hallucinations. Psychological Medicine.

[bb0065] Daalman K., Verkooijen S., Derks E.M., Aleman A., Sommer I.E.C. (2012). The influence of semantic top-down processing in auditory verbal hallucinations. Schizophrenia Research.

[bb9015] Daalman K., Sommer I.E.C., Derks E.M. (2013). Cognitive biases and auditory verbal hallucinations in healthy and clinical individuals. Psychological Medicine.

[bb0070] Daalman K., Diederen K.M.J., Hoekema L., van Lutterveld R., Sommer I.E.C. (2016). Five year follow-up of non-psychotic adults with frequent auditory verbal hallucinations: Are they still healthy?.

[bb0075] Daskalakis N.P., Bagot R.C., Parker K.J., Vinkers C.H., de Kloet E.R. (2013). The three-hit concept of vulnerability and resilience: Toward understanding adaptation to early-life adversity outcome. Psychoneuroendocrinology.

[bb0080] David A. (2010). Why we need more debate on whether psychotic symptoms lie on a continuum with normality. Psychological Medicine.

[bb0085] Davies M.F., Griffin M., Vice S. (2001). Affective reactions to auditory hallucinations in psychotic, evangelical and control groups. The British Journal of Clinical Psychology/the British Psychological Society.

[bb0090] de Leede-Smith S., Barkus E. (2013). A comprehensive review of auditory verbal hallucinations: Lifetime prevalence, correlates and mechanisms in healthy and clinical individuals. Frontiers in Human Neuroscience.

[bb0095] De Loore E., Gunther N., Drukker M., Feron F., Sabbe B., Deboutte D., Myin-Germeys I. (2011). Persistence and outcome of auditory hallucinations in adolescence: A longitudinal general population study of 1800 individuals. Schizophrenia Research.

[bb0100] De Weijer A.D., Neggers S.F.W., Diederen K.M.S., Mandl R.C.W., Kahn R.S., Hulshoff Pol H.E., Sommer I.E. (2013). Aberrations in the arcuate fasciculus are associated with auditory verbal hallucinations in psychotic and in non-psychotic individuals. Human Brain Mapping.

[bb0110] Diederen K.M.J., De Weijer A.D., Daalman K., Blom J.D., Neggers S.F.W., Kahn R.S., Sommer I.E.C. (2010). Decreased language lateralization is characteristic of psychosis, not auditory hallucinations. Brain.

[bb0115] Diederen K.M.J., Daalman K., De Weijer A.D., Neggers S.F.W., Van Gastel W., Blom J.D., Sommer I.E.C. (2012). Auditory hallucinations elicit similar brain activation in psychotic and nonpsychotic individuals. Schizophrenia Bulletin.

[bb0120] Diederen K.M.J., Neggers S.F.W., de Weijer a.D., van Lutterveld R., Daalman K., Eickhoff S.B., Sommer I.E.C. (2013). Aberrant resting-state connectivity in non-psychotic individuals with auditory hallucinations. Psychological Medicine.

[bb9020] Fleming M.P., Martin C.R. (2009). A preliminary investigation into the experience of symptoms of psychosis in mental health professionals: Implications for the psychiatric classification model of schizophrenia. Journal of Psychiatric and Mental Health Nursing.

[bb0125] Fusar-Poli P., Meyer-Lindenberg A. (2013). Striatal presynaptic dopamine in schizophrenia, part II: Meta-analysis of [18F/11C]-DOPA PET studies. Schizophrenia Bulletin.

[bb0130] Hill K., Varese F., Jackson M., Linden D.E.J. (2012). The relationship between metacognitive beliefs, auditory hallucinations, and hallucination-related distress in clinical and non-clinical voice-hearers. British Journal of Clinical Psychology.

[bb0135] Honig A., Romme M., Ensink B.J., Escher S.D., Pennings M.H., deVries M.W. (1998). Auditory hallucinations: A comparison between patients and nonpatients. The Journal of Nervous and Mental Disease.

[bb0140] Howes O.D., Kapur S. (2009). The dopamine hypothesis of schizophrenia: Version III - The final common pathway. Schizophrenia Bulletin.

[bb0145] Howes O.D., Bose S.K., Turkheimer F., Valli I., Egerton A., Valmaggia L.R., McGuire P. (2011). Dopamine synthesis capacity before onset of psychosis: A prospective [18F]-DOPA PET imaging study. American Journal of Psychiatry.

[bb9025] Howes O.D., Kambeitz J., Kim E. (2012). The nature of dopamine dysfunction in schizophrenia and what this means for treatment. Archives of General Psychiatry.

[bb0150] Howes O.D., Shotbolt P., Bloomfield M., Daalman K., Demjaha A., Diederen K.M.J., Sommer I.E. (2013). Dopaminergic function in the psychosis spectrum: An [18F]-DOPA imaging study in healthy individuals with auditory hallucinations. Schizophrenia Bulletin.

[bb0155] Howes O.D., Williams M., Ibrahim K., Leung G., Egerton A., McGuire P.K., Turkheimer F. (2013). Midbrain dopamine function in schizophrenia and depression: A post-mortem and positron emission tomographic imaging study. Brain: A Journal of Neurology.

[bb9030] Howes O.D., McCutcheon R., Owen M.J. (2016). The role of genes, stress and dopamine in the development of schizophrenia. Biological Psychiatry Published Online First.

[bb0160] Insel T., Cuthbert B., Garvey M., Heinssen R., Pine D.S., Quinn K., Wang P. (2010). Research domain criteria (RDoC): Toward a new classification framework for research on mental disorders. American Journal of Psychiatry.

[bb0165] Jacobsen, P., Peters, E., Ward, T., Garety, P.A., Jackson, M., & Chadwick, P., (Manuscript submitted) Overgeneral Autobiographical Memory Bias in Clinical and Non-Clinical Voice-Hearers. (Received via personal communication) 2016, (unpublished). 10.1017/S0033291718000570 PMC6004309 29536827

[bb0170] Johns L.C., Kompus K., Connell M., Humpston C., Lincoln T.M., Longden E., Larøi F. (2014). Auditory verbal hallucinations in persons with and without a need for care. Schizophrenia Bulletin.

[bb0175] Kaymaz N., van Os J. (2010). Extended psychosis phenotype–yes: Single continuum–unlikely. Psychological Medicine.

[bb9035] Kaymaz N., Drukker M., Lieb R., Wittchen H.U., Werbeloff N., Weiser M., van Os J. (2012). Do subthreshold psychotic experiences predict clinical outcomes in unselected nonhelp-seeking population-based samples? A systematic review and meta-analysis, enriched with new results. Psychological Medicine.

[bb0180] Kompus K., Falkenberg L.E., Bless J.J., Johnsen E., Kroken R.A., Kråkvik B., Hugdahl K. (2013). The role of the primary auditory cortex in the neural mechanism of auditory verbal hallucinations. Frontiers in Human Neuroscience.

[bb0185] Kråkvik B., Larøi F., Kalhovde A.M., Hugdahl K., Kompus K., Salvesen Ø., Vedul-Kjelsås E. (2015). Prevalence of auditory verbal hallucinations in a general population: A group comparison study. Scandinavian Journal of Psychology.

[bb0190] Larøi F. (2012). How do auditory verbal hallucinations in patients differ from those in non-patients?. Frontiers in Human Neuroscience.

[bb0195] Launay G., Slade P.D. (1981). The measurement of hallucinatory predisposition in male and female prisoners. Personality and Individual Diåerences.

[bb0200] Lawrence C., Jones J., Cooper M. (2010). Hearing voices in a non-psychiatric population. Behavioural and Cognitive Psychotherapy.

[bb0205] Leudar I., Thomas P., McNally D., Glinski A. (1997). What voices can do with words: Pragmatics of verbal hallucinations. Psychological Medicine.

[bb0210] Linden D.E.J., Thornton K., Kuswanto C.N., Johnston S.J., Van De Ven V., Jackson M.C. (2011). The brain's voices: Comparing nonclinical auditory hallucinations and imagery. Cerebral Cortex.

[bb0215] Linscott R.J., van Os J. (2010). Systematic reviews of categorical versus continuum models in psychosis: Evidence for discontinuous subpopulations underlying a psychometric continuum. Implications for DSM-V, DSM-VI, and DSM-VII. Annual Review of Clinical Psychology.

[bb0220] Linscott R.J., van Os J. (2013). An updated and conservative systematic review and meta-analysis of epidemiological evidence on psychotic experiences in children and adults: On the pathway from proneness to persistence to dimensional expression across mental disorders. Psychological Medicine.

[bb9040] McGrath J.J., Saha S., Al-Hamzawi A., Alonso J., Bromet E.J., Bruffaerts R., Kessler R.C. (2015). Psychotic Experiences in the General Population: A Cross-National Analysis Based on 31,261 Respondents From 18 Countries. JAMA Psychiatry.

[bb0225] Moritz S., Larøi F. (2008). Differences and similarities in the sensory and cognitive signatures of voice-hearing, intrusions and thoughts. Schizophrenia Research.

[bb0230] Peters E., Ward T., Jackson M., Morgan C., Charalambides M., McGuire P., Garety P.A. (2016). Clinical, socio-demographic and psychological characteristics in individuals with persistent psychotic experiences with and without a “need for care.”. World Psychiatry.

[bb0235] Read J., Van Os J., Morrison A.P., Ross C.a. (2005). Childhood trauma, psychosis and schizophrenia: A literature review with theoretical and clinical implications. Acta Psychiatrica Scandinavica.

[bb0240] Russo M., Levine S.Z., Demjaha A., Di Forti M., Bonaccorso S., Fearon P., Reichenberg A. (2014). Association between symptom dimensions and categorical diagnoses of psychosis: A cross-sectional and longitudinal investigation. Schizophrenia Bulletin.

[bb0245] Schneider K. (1959). Clinical psychopathology.

[bb0250] Shevlin M., Houston J.E., Dorahy M.J., Adamson G. (2008). Cumulative traumas and psychosis: An analysis of the national comorbidity survey and the British Psychiatric Morbidity Survey. Schizophrenia Bulletin.

[bb9045] Slotema C.W., Daalman K., Blom J.D. (2012). Auditory verbal hallucinations in patients with borderline personality disorder are similar to those in schizophrenia. Psychological Medicine.

[bb9050] Smailes D., Alderson-Day B., Fernyhough C. (2015). Tailoring Cognitive Behavioral Therapy to Subtypes of Voice-Hearing. Frontiers in Psychology.

[bb0255] Sommer I.E., Daalman K., Rietkerk T., Diederen K.M., Bakker S., Wijkstra J., Boks M.P.M. (2010). Healthy individuals with auditory verbal hallucinations; who are they? Psychiatric assessments of a selected sample of 103 subjects. Schizophrenia Bulletin.

[bb0260] Sommer I.E., Derwort a.M.C., Daalman K., de Weijer A.D., Liddle P.F., Boks M.P.M. (2010). Formal thought disorder in non-clinical individuals with auditory verbal hallucinations. Schizophrenia Research.

[bb0265] Sorrell E., Hayward M., Meddings S. (2010). Interpersonal processes and hearing voices: A study of the association between relating to voices and distress in clinical and non-clinical hearers. Behavioural and Cognitive Psychotherapy.

[bb0270] Taylor G., Murray C. (2012). A qualitative investigation into non-clinical voice hearing: What factors may protect against distress? Mental health. Religion & Culture.

[bb9055] Upthegrove R., Broome M.R., Caldwell K. (2016). Understanding auditory verbal hallucinations: a systematic review of current evidence. Acta Psychiatrica Scandinavica.

[bb0275] van Lutterveld R., Oranje B., Kemner C., Abramovic L., Willems A.E., Boks M.P.M., Sommer I.E.C. (2010). Increased psychophysiological parameters of attention in non-psychotic individuals with auditory verbal hallucinations. Schizophrenia Research.

[bb0280] van Lutterveld R., van den Heuvel M.P., Diederen K.M.J., de Weijer A.D., Begemann M.J.H., Brouwer R.M., Sommer I.E. (2014). Cortical thickness in individuals with non-clinical and clinical psychotic symptoms. Brain: A Journal of Neurology.

[bb0285] Van Os J. (2009). A salience dysregulation syndrome. British Journal of Psychiatry.

[bb9060] van Os J., Reininghaus U. (2016). Psychosis as a transdiagnostic and extended phenotype in the general population. World Psychiatry.

[bb0290] van Os J., Linscott R.J., Myin-Germeys I., Delespaul P., Krabbendam L. (2009). A systematic review and meta-analysis of the psychosis continuum: Evidence for a psychosis proneness-persistence-impairment model of psychotic disorder. Psychological Medicine.

[bb0295] Varese F., Smeets F., Drukker M., Lieverse R., Lataster T., Viechtbauer W., Bentall R.P. (2012). Childhood adversities increase the risk of psychosis: A meta-analysis of patient-control, prospective-and cross-sectional cohort studies. Schizophrenia Bulletin.

[bb0300] Varese F., Tai S.J., Pearson L., Mansell W. (2016). Thematic associations between personal goals and clinical and non-clinical voices (auditory verbal hallucinations). Psychosis.

[bb0305] Wenzlaff R.M., Wegner D.M. (2000). Thought suppression. Annual Review of Psychology.

[bb0310] Wigman J.T.W., Lin A., Vollebergh W.A., van Os J., Raaijmakers Q.a.W., Nelson B., Yung a.R. (2011). Subclinical psychosis and depression: Co-occurring phenomena that do not predict each other over time. Schizophrenia Research.

[bb0315] Wigman J.T.W., Vollebergh W.A., Raaijmakers Q.A., Iedema J., Van Dorsselaer S., Ormel J., Van Os J. (2011). The structure of the extended psychosis phenotype in early adolescence - A cross-sample replication. Schizophrenia Bulletin.

[bb0320] Woods A., Jones N., Alderson-Day B., Callard F., Fernyhough C. (2015). Experiences of hearing voices: Analysis of a novel phenomenological survey. The Lancet Psychiatry.

